# Suicides as a response to adverse market sentiment (1980-2016)

**DOI:** 10.1371/journal.pone.0186913

**Published:** 2017-11-02

**Authors:** Pankaj Agrrawal, Doug Waggle, Daniel H. Sandweiss

**Affiliations:** 1 MaineBusiness School, University of Maine, Orono, Maine, United States of America; 2 Department of Accounting and Finance, University of West Florida, Pensacola, Florida, United States of America; 3 Department of Anthropology, University of Maine, Orono, Maine, United States of America; 4 Climate Change Institute, Bryand Global Sciences Center, University of Maine, Orono, Maine, United States of America; Yokohama City University, JAPAN

## Abstract

Financial crises inflict significant human as well as economic hardship. This paper focuses on the human fallout of capital market stress. Financial stress-induced behavioral changes can manifest in higher suicide and murder-suicide rates. We find that these rates also correlate with the Gross Domestic Product (GDP) growth rate (negatively associated; a -0.25% drop [in the rate of change in annual suicides for a +1% change in the independent variable]), unemployment rate (positive link; 0.298% increase), inflation rate (positive link; 0.169% increase in suicide rate levels) and stock market returns adjusted for the risk-free T-Bill rate (negative link; -0.047% drop). Suicides tend to rise during periods of economic turmoil, such as the recent Great Recession of 2008. An analysis of Centers for Disease Control and Prevention (CDC) data of more than 2 million non-natural deaths in the US since 1980 reveals a positive correlation with unemployment levels. We find that suicides and murder-suicides associated with adverse market sentiment lag the initial stressor by up to two years, thus opening a policy window for government/public health intervention to reduce these negative outcomes. Both our models explain about 73 to 76% of the variance in suicide rates and rate of change in suicide rates, and deploy a total of four widely available independent variables (lagged and/or transformed). The results are invariant to the inclusion/exclusion of 2008 data over the 1980–2016 time series, the period of our study. The disconnect between rational decision making, induced by cognitive dissonance and severe financial stress can lead to suboptimal outcomes, not only in the area of investing, but in a direct loss of human capital. No economic system can afford such losses. Finance journal articles focus on monetary alpha, which is the return on a portfolio in excess of the benchmark; we think it is important to be aware of the loss of human capital as a consequence of market instability. This study makes one such an attempt.

## Introduction

The negative consequences of economic recessions and depressions include fatal violence, both suicides and homicides. For every homicide in the U.S., there are, on average, 1.97 reported suicides (supporting information S1 Table). The incidence of suicide, however, is not invariant. We explored the relation of suicide, homicide, and murder-suicide with the financial crisis triggered by the collapse of the housing market during the Great Recession of 2008 [1]. A murder-suicide, or suicide-homicide, is a specific crime in which a homicide is followed by the perpetrator committing suicide [2]. The Center for Disease Control’s National Violent Death Reporting System (CDC/NVDRS) data classifies murder-suicide (homicide-suicide) as a separate entity. It is not the sum of all homicides and suicides committed independently.

Norström and Grönqvist [3] document the unemployment-suicide link during the Great Recession, when economic sentiment was persistently adverse, and find that it is favorably modified by system-based supportive measures. Behavioral and cognitive alteration during financial crisis is correlated with stress and suicide rates, as explored in the work of Mucci *et al*. [4]. Jones and Pridemore [5] also support a link between the US housing crisis of 2008 and race-specific suicide rates. The traditional causation directionality is that behavior and investor sentiment impacts financial valuation [6]. Here, we explore the implication of valuation turmoil on severe behavioral instability, eventually resulting in loss of value.

Increased occurrence of these fatal events, or “direct human fallout” (our term), is tightly correlated with the disruption caused by economic, financial, and social hardship linked to the global financial crisis (GFC). Coope *et al*. [7] identify variables other than unemployment, such as house repossessions to contribute to increased suicide rates. In this paper, we test the hypotheses that the global financial crisis (GFC) and the associated adverse economic sentiment, triggered by the collapse of the housing market in 2008, would increase suicide, homicide, and murder-suicide rates. We found that to be the case and also find that direct human fallout lags the triggering events by up to two years, thus opening an interventionist policy space for enhanced prevention of potential capital loss—both human and physical. Distressful as it may be, it is possible to quantify, to a certain extent, the capital loss from suicide and murder-suicide. For instance, the value of a statistical year of life implied by dialysis practice currently averages $129,090 per year [8].

## Materials and methods

We investigated the occurrence of suicide, homicide, and murder-suicide (where the perpetrator commits suicide within one week of the murder) as the final breakdown of an individual facing adversity resulting from foreclosures, mortgage delinquencies, evictions, and other financial hardships associated with the GFC. We also explored the association of these suicides and homicides with economic and capital market activity (as proxied by a rise in US unemployment levels, fluctuating GDP growth rates, inflation rates, and stock market returns, Tables 1 and 2; the associated regression scatter plots can be seen in the supporting information S3 Fig). In the body of the paper we also show the higher incidence of suicides in the years 2008, 2009 and 2010 and further break it down by events where the known condition was a job or financial problem (Table 3).

**Table 1:** Suicides: Model #1 OLS regression between the lagged US unemployment rate, inflation rate, GDP growth rate and CDC- provided annual suicide rates (Y-variable). All p-values are significant at the 0.015 level. The overall regression F-stat is 23.48 and significant at the 0.015 p-value. The Durbin-Watson test value of 0.6478 indicates a slight positive autocorrelation in the regression residuals. The adjusted R^2^ indicates that 69.93% of the variation in the suicide rate is explained by the three independent variables. Annual rates are since 1980. Data from [9], [10].

Y = SUIC_t_	Coeff.	Std. Err.	t-stat	p-value	F(3,26) 23.4767 ***	p- (regrsn) 1.44E-07***
constant	9.39943				R^2^ 0.7304	Adjusted R^2^ 0.6993
UNEMP_t-2_	0.332397	0.048896	6.7981	<0.00001		
INFL_t-1_	0.169057	0.063175	2.6760	0.01272		
GDP_t-1_	-0.109126	0.039570	-2.7578	0.01051		Durbin-Watson 0.6478

*** indicates significant at p < 0.0001

**Table 2:** Change in suicide rate: Model #2 OLS regression between the market risk premium (STOCK_t_), the unemployment rate (UNEMP_t_), the real GDP growth rate (GDP_t_) and rate of change in annual suicides (Y-variable). The overall regression F-stat is 30.69 and significant at the 0.001 p-value. The Durbin-Watson test value of 2.1038 indicates no autocorrelation in the regression residuals. The adjusted R^2^ indicates that 74.18% of the variation in the change in suicide rate is explained by the three independent variables. Annual rates are since 1980. Data from [9], [10].

Y = ΔSUIC_t_	Coeff.	Std. Err.	t-stat	p-value	F(3,28) 30.6855 ***	p- (regrsn) 5.40E-09***
constant	-0.948493				R^2^ 0.76678	Adjusted R^2^ 0.74179
GDP_t_	-0.254423	0.10969	-2.3195	0.02788		
UNEMP_t_	0.298366	0.168572	1.7700	0.08762		
STOCK_t_	-0.0473968	0.00774383	-6.1206	<0.00001		Durbin-Watson 2.1038

*** indicates significant at p<0.0001

**Table 3:** Suicides data from the National Violent Death Reporting System (2005–2016*). The sixteen states participating in the NVDRS (of the CDC) are Alaska, Colorado, Georgia, Kentucky, Maryland, Massachusetts, New Jersey, New Mexico, North Carolina, Oklahoma, Oregon, Rhode Island, South Carolina, Utah, Virginia, and Wisconsin. Note the higher suicide incidence rates for the years 2008, 2009 and 2010 [3]. The data is available up to 12/2013, but recorded as of 10/2016, due to reporting lags. The last row in Table 3 also shows the percentage of suicides triggered by financial problems affecting the agent. Information similar to Table 2 shows the occurrence of murder-suicides as a joint event and can be found in the supporting information S2 Table.

Total Suicides									
Year	2005	2006	2007	2008	2009	2010	2011	2012	2013
Male	6843	6719	7205	7400	7827	7974	8139	8374	8711
Female	1850	1878	2027	2070	2122	2201	2328	2474	2533
TOTAL	8694	8597	9233	9471	9949	10176	10467	10848	11244
**Total Suicides per 100,000 People**									
Male	17.95	17.38	18.42	18.69	19.56	19.78	19.97	20.37	21.02
Female	4.67	4.68	4.99	5.03	5.10	5.26	5.51	5.81	5.90
TOTAL	**11.16**	**10.91**	**11.57**	***11*.*72***	***12*.*19***	***12*.*38***	***12*.*61***	***12*.*96***	***13*.*33***
**Suicides with Firearms**									
Male	4029	3834	4044	4249	4437	4366	4709	4853	4948
Female	579	591	651	651	720	650	784	764	825
TOTAL	4608	4425	4695	4900	5157	5016	5493	5617	5773
**Suicides with Firearms per 100,000 People**									
Male	10.53	9.88	10.29	10.69	11.04	10.83	11.56	11.81	11.94
Female	1.46	1.47	1.60	1.58	1.73	1.55	1.85	1.79	1.92
TOTAL	5.91	5.61	5.87	6.06	6.31	6.10	6.62	6.71	6.84
**Percentages of Events Where Conditions / Location Known**									
Job problem*	*11*.*21*	*11*.*29*	*11*.*48*	13.4	14.63	15.37	13.99	12.68	12.4
Financial problem*	*11*.*20*	*11*.*77*	*11*.*69*	***13*.*41***	***13*.*76***	***13*.*55***	11.98	10.8	10.95
Eviction or loss of home **						4.18	4.31	3.59	3.77

* Events may be included in multiple categories.

** Category added in 2010. Figures from earlier years considered unreliable.

We analyzed over 2.79 million injury-related deaths to isolate suicides (supporting information S1 Table) and national suicide rates for the past three decades. This paper also extends the Eliason study [2], which tabulated the incidence of ‘murder-suicide’ events up to 2004, to include the period from 2005 through 2013 (supporting information S3 Table). The National Vital Statistics System of the Centers for Disease Control and Prevention [10] provides data on the annual suicide rates from 1980 to 2014 (CDC data trail real time by two years). Here, we present two multivariate lagged models, thus extending the fixed rate mortality-unemployment model of Stuckler *et al*. [11]. These suicide rates are linked to the US unemployment rate, the GDP growth rate, inflation rate, and stock market returns adjusted for the risk-free T-Bill rate [9], Bureau of Economic Analysis [12], and Center for Research in Security Prices [13].

The collapse of the housing sector began towards the end of 2006. About 2.5 million foreclosures took place within just three years. Treuhaft *et al*. [14] estimated in 2012 that about 10 million more foreclosures would occur before the crisis abated. This was the largest spike in foreclosures since the Great Depression of 1929. Bernanke [15] noted in 2012 that about 12 million mortgages were underwater with an aggregate negative equity of $700 billion. The Economic Policy Institute’s Allegretto [16] estimated that US families had already lost about six trillion dollars of personal wealth during the crisis from 2007 to 2010, equivalent to 39% of the US national GDP in 2011 [12]).

The loss or threat of loss of a home can be extremely destabilizing for individuals, especially those with limited safety nets, such as the 46% of US households with less than $5,000 of liquid assets [17]. Families facing foreclosure experience high rates of depression, marital discord, and declines in academic performance [18]. The forced relocations often have a disproportionately adverse effect on children and seniors who find themselves uprooted from their social networks and transplanted into unfamiliar settings. Such levels of stress can initiate a vicious cycle of health problems, mental issues, and loss of earnings [19]. In the last half of 2007 about 28 percent of all mortgage delinquencies and foreclosures were attributed to household heads age 50 and older, a group with a foreclosure rate double the national average [20].

## Data

This study utilized data from three external sources (as of October 2016): (1) annual suicide rate series from 1980 to 2014 [10]; (2) detailed suicide and murder-suicide rates for the period 2005–2013 ([21]); and (3) the US annual civilian unemployment rate for the period 1980 through 2016 [9], [22]. It may be noted that the murder-suicide dataset in [21] trails real time data by three years and it only compiles information from sixteen US states, while the CDC suicide data lags has a reporting lag of two years.

## Results

While transportation-related deaths are the single highest cause of unnatural, non-disease deaths (25% of the total), the CDC reports that there are almost twice as many suicides as homicides (20% versus 10%) (supporting information S1 Table). From 1999–2014, about 509 injury-related deaths occurred per day in the United States, of which about 102 per day resulted from suicides.

Table 3 and supporting information S2 Table, present mortality suicide data for a sixteen-state subset of the U.S. [21] with two refinements: rates for ‘finance-related stressors’ that triggered the suicides, and ‘murder-suicides’ as a distinct category ([2]. The suicide rate over the period 2005–2007 averages 11.21 per 100,000, but for the years 2008 through 2013, the average suicide rate climbed to 12.53 per 100,000 (Table 3, data as of 10/2016, averages not shown, significant at p <0.05). Likewise, suicides resulting from a ‘financial problem’ rose from 11.55% of instances from 2005–2007 to 12.41% of instances from 2008–2013. The total percentage of joint murder-suicides known to be triggered by a ‘financial problem’ peaked at 10.98% during 2009, while the average for the entire 2005–2013 period was 7.56% (supporting information S2 Table). It was during 2008 that the US equity market as proxied by the S&P 500 lost about 38% (it was actually down 55% from the October 2007 peak to the March 2009 trough) of its value [13] resulting in severe erosion of value in retirement and brokerage accounts and compression in real estate valuations. The effect of such fast and large declines is compounded for individuals who are leveraged, either in their equity or real estate portfolios, resulting in personal and social disruption as well. Referring to the supporting information S1 Fig, it can be seen that the suicide rate climbed in the 2005–2014 period to about 12.25 (per 100,000 individuals) compared to 10.75 in the prior five years (p < 0.01). During the 2007–2010 period, the unemployment rate went up from about 5.1% to 9.6%. Interestingly, since 2007, the labor force participation rates have been dropping steadily, a relatively new phenomenon ([9] *timeseries*: http://data.bls.gov/timeseries/LNS11300000). Labor force participation dropped from about 66% in 2007 to 63% in 2016, resulting in a drop in the unemployment rate, but with a continuous increase in suicide rates. The hardship for those who have entirely given up on job searches and pulled out of the labor force possibly manifests in the steadily climbing suicide rate, despite an apparent decline in unemployment rates post 2010 (supporting information S2 Fig).

We developed two models which extend the fixed rate mortality-unemployment model of Stuckler *et al*. [11], who used European data later reported in Arora et al. [23], to assess the link between suicides and proxies for economic activity, one for the aggregate level of ‘suicide rates’ and the other for the annual ‘rate of change’ in suicide rates, year over year. We selected a parsimonious set of independent variables that also made intuitive sense, were easily observable, and did not require massively complex transformations that would make subsequent application or interpretation cumbersome. The significant explanatory variables were the unemployment rate, the GDP growth rate, the inflation rate, and the market risk premium; the first three variables are lagged (Tables 1 and 2).

With Model #1 (Table 1) we determined the link between the CDC reported (CDC, [2016]) annual suicide rates (SUIC_t_) and the corresponding two-year-lagged unemployment rate (UNEMP_t-2_), lagged inflation rate (INFL_t-1_) and the lagged real GDP growth rate (GDP_t-1_). The three explanatory variables, when in adverse states, are proxies for general economic malaise. The variables were checked for any collinearity issues using the Variance Inflation Factor test (VIF(j) = 1/(1 − R(j)^2^), where R(j) is the multiple correlation coefficient between variable j and the other independent variables.) All variables had values around 1.1, well below the threshold value of 10. The functional form of our first model is:

$${SUIC}_{t}=\varphi ({UNEMP}_{t-2},{INFL}_{t-1},{GDP}_{t-1},e_{t})$$

where t is the year, e the error term and the other variables are as explained in the preceding paragraph.

Table 1 shows the OLS regression coefficients between the lagged US unemployment rate, inflation rate, GDP growth rate, and annual suicide rates (Y-variable) from 1980. We used the hetereoscedasticity-adjusted OLS regression variant, which is essentially a two-step WLS regression, with weights of the reciprocal of the estimated variance. The overall regression F-stat of 23.48 is significant with a p-value below 0.001. The Durbin-Watson test value of 0.6478 indicates a slight positive autocorrelation in the regression residuals. The adjusted R^2^ indicates that 69.93% of the variation in the suicide rate is explained by the three independent variables. All p-values for the independent variables are significant at the 0.015 level. The strongest covariate is the lagged US unemployment rate (t-stat = 6.7981) for which we find a Pearson-correlation of 0.61 (p<0.001) with suicide rates. This also corresponds well with a long-term business cycle and age-specific study of suicide rates [24], which found a correlation of about 0.61 for the 45–64 age group, with contemporaneous variables. A 95% ellipse of the scatter points between the suicide rate (X-axis) and the t-2 year lagged unemployment rate (Y axis) (supporting information S2 Fig) confirms the strong positive association between the two variables, also brought out in the regression coefficients (Table 1).

Based on the numerical output of the regression coefficients, the following covariate effects emerge:

∂SUICt/∂UNEMPt-2>0; higher past unemployment rates (t-2) lead to increased current suicide rates (p-value <0.00001)

∂SUICt/∂INFLt-2>0; higher past inflation rates (t-1) lead to increased current suicide rates (p-value <0.01)

∂SUICt/∂GDPt-2>0; lower past GDP growth rates (t-1) lead to higher current suicide rates (p-value <0.01)

The second model (Table 2) isolates the association between the rate of change of the annual suicide rate (ΔSUIC_t_) and three un-lagged explanatory variables—the market risk premium (STOCK_t_) (the difference between the stock market return (S&P 500) and the risk-free T-Bill rate is the market risk premium; in normal states it is often positive, but it can become negative during periods of stock market declines), the unemployment rate (UNEMP_t_), and the real GDP growth rate (GDP_t_). The functional form of our second model is thus:

$${\Delta SUIC}_{t}=\varphi ({{STOCK}_{t},UNEMP}_{t},{GDP}_{t},e_{t})$$

where t is the year and e the error term (the Durbin-Watson test {2.1038} indicated no autocorrelation in the regression, Table 2). Based on the numerical output of the regression coefficients, the following covariate effects emerge for the second model:

∂ΔSUICt/∂UNEMPt-2>0; higher unemployment rates lead to increased year-over-year change in suicide rates at time (t) and vice versa (p-value <0.09)

∂ΔSUICt/∂STOCKt-2>0; lower stock market returns lead to higher year-over-year change in suicide rates at time (t) and vice versa (p-value <0.00001)

∂ΔSUICt/∂GDPt-2>0; lower GDP growth rates lead to higher year-over-year change in suicide rates at time (t) and vice versa (p-value <0.03)

For this multivariate regression, the adjusted R^2^ was 74.18% (p-value of the F-test <0.001, Table 2). The strongest covariate was the stock market risk premium (STOCK_t_) with a t-stat of -6.1206, indicative of a rise in suicide rates in negative capital market environments. The higher incidence of murder-suicides, suicides, and homicides in the post-2008 period is also noted earlier in this study (Table 3 and supporting information S2 Table), with data on all injury-related deaths from 1999 to 2014 [25]. The Variance Inflation Factor test indicated no notable collinearity within the independent regressor variables.

Table 2 shows the results of the OLS regression between the the market risk premium (STOCK_t_), the unemployment rate (UNEMP_t_), the real GDP growth rate (GDP_t_) and rate of change in annual suicides (Y-variable). The overall regression F-stat is 30.69 and significant at the 0.001 p-value. The Durbin-Watson test value of 2.1038 indicates no autocorrelation in the regression residuals. The adjusted R^2^ indicates that 74.18% of the variation in the change in suicide rate is explained by these three independent variables.

## Discussion/Conclusions

As noted in the pioneering work of Catalano [26]) and Stuckler *et al*. [27], it takes time for economic hardship to work its way through the socio-economic system and ultimately disrupt the psychiatric state of the affected individual to such an extent that it leads to their violent and untimely death. The suicide rate climbed in the 2005–2014 period to about 12.25 persons compared to 10.75 persons per 100,000 population in the previous five years, when unemployment was rising (supporting information S1 Fig, [9, 10]). As mentioned earlier, unemployment rates have declined since 2010 but that could be due to declining labor force participation rates, from about 66% in 2007 to 63% in 2016 (supporting information S2 Fig, [9] *timeseries*: *LNS11300000*), yet the suicide rate continues to rise (supporting information S1 Fig). The so-called Great Recession of 2008 comes closest to the Great Depression of 1929 with regard to its impact and severity; in both instances, the suicide rates peaked about two years from the low point in the capital markets. It is perhaps a testimony to modern social support mechanisms that the suicide rate is still far below the 22.1 per 100,000 rate seen in 1932 [24].

Nevertheless, as Reeves *et al*. [28] cogently argue, the increase in suicides associated with severe economic crisis calls for enhanced anticipatory policy intervention to lower the suicide/homicide rates, a result echoed by Chan *et al*. [29] in their South Korean study of diverse populations. Chen *et al*. [30] find stock market declines to result in neurotic behavioral disorders and increased health care access visits. Research by Stack [31, 32] provides a review of various time series studies of suicide going back to the 1930’s and finds that migration (which lowers social integration) and economic strain have a positive link with suicide. The evidence from that period is mixed in the sense that omitting the severe economic depression of the 1930’s leads to a weakening of the link between unemployment and suicides. This could be also somewhat buffered by the high levels of government pump priming following the great Depression of 1929, where the potential continuation of the higher suicide rate was reduced as a result of governmental active intervention in the labor market in 1933. Nonetheless, we excluded all data from 2008 (The Great Recession year) and find that results are invariant to the change (OLS Model 1 and Model 2), in fact the reduction in volatility introduced by the 2008 data results in the R^2^ of both the models to marginally go up. Stack and Laubepin [33] also note that while economic “development fosters internal locus of control, the link between development and direction of violence is not explained by locus of control.” In our paper, it appears that shocks from economic and financial stress manifest as a direct loss of human capital, which to a certain extent can be mitigated by anticipatory coordinated intervention at the macro and public governmental level.

The two-year lag between job loss and eventual suicide offers a ‘window of opportunity’ for such actions, where the disconnect between rational decision making, induced by cognitive dissonance and severe financial stress, can lead to suboptimal outcomes, not only in the area of investing but in a direct loss of human capital. No economic system can afford such losses. This paper seeks to raise the awareness of decision makers to this ‘human fallout’ with the intention that strategy be formulated to prevent such negative outcomes of financial meltdowns that are bound to recur and disturb the human ecosystem, social diaspora and the financial landscape.

## Supporting information

S1 Text Supporting references. (DOCX)

S1 Fig Unemployment rate versus suicide rate (1980–2016*). The correlation of these series is 0.62 (p-value <0.001). The suicide rate climbed in the 2005–2014 period to about 12.25 compared to 10.75 in the prior five years. During 2007–2010 the unemployment rate went up from about 5.1% to 9.6%. Since 2007, the Labor force participation rates have been dropping steadily, a relatively new phenomenon, from about 66% to 63% in 2016, resulting in a drop in the unemployment rate. The hardship for those who have given up on job searches possibly manifests in the steadily climbing suicide rate, post 2010. Data from [10], [9]: timeseries: LNS11300000, (*data available is only up to 12/2014, *but as of 10/2016*).
(DOCX)

S2 Fig Declining labor force participation. Since 2007, the labor force participation rates have been dropping steadily, a relatively new phenomenon, from about 66% to 63% in 2016, resulting in a drop in the unemployment rate. The hardship for those who have given up on job searches possibly manifests in the steadily climbing suicide rate, post 2010. Data from [9]: *timeseries*:*LNS11300000*.
(DOCX)

S3 Fig OLS regression model: Suicide rate. SUIC_t_ = *φ*(UNEMP_t-2_, INFL_t-1_, GDP_t-1_, e_t_) This figure shows two aspects of the OLS regression with the CDC provided annual suicide rates as the dependent variable (all p-values are significant at the 0.015 level, Table 1). The first exhibit indicates a positive relationship between the lagged national unemployment rate and the incidence of suicides. A 95% ellipse is also shown around the x-y points. The second exhibit shows the estimated versus the actual suicide rates. The observed adjusted R^2^ of this regression is 69.93%. Annual rates are since 1980. From [9], [10], [25].
(DOCX)

S1 Table Distribution of 2.79 Million U.S. Injury-Related Deaths (1999–2016*). Compiled and processed from [21] (*data available is only up to 12/2014, *but as of 10/2016*).
(DOCX)

S2 Table Murder-Suicide (a joint event) data from National Violent Death Reporting System (2005–2016*). The sixteen states participating in the NVDRS (of CDC) are Alaska, Colorado, Georgia, Kentucky, Maryland, Massachusetts, New Jersey, New Mexico, North Carolina, Oklahoma, Oregon, Rhode Island, South Carolina, Utah, Virginia, and Wisconsin. Note the higher incidence rates for joint murder-suicides in 2009. Murder-Suicide is a separate crime class by itself and is not the sum of murders and suicides. Note the higher than average incidence rates for the years 2009 and 2010 [21] (*data available is only up to 12/2013, *but as of 10/2016)*.
(DOCX)

S3 Table Overview of previous studies on murder-suicide**. Eliason [2] presents information on studies that report the incidence of 640 joint murder-suicides over the period 1980–2004. This study includes an additional 1680 such deaths for the period 2005–2013 (** *available as of 10/2016*).
(DOCX)
