# Supplementary material for: Suicides as a response to adverse market sentiment (1980-2016)
Source: PLoS One. 2017 Nov 2;12(11):e0186913. doi: 10.1371/journal.pone.0186913 (PMC5667934; doi:10.1371/journal.pone.0186913)
Supplement: S1 Text — (DOCX) [file pone.0186913.s001.docx]

**S1 Text Supporting References**

1. Centers for Disease Control and Prevention (CDC). National Vital Statistics System [Data file]; 2016. Retrieved from http://www.cdc.gov/nchs/nvss.htm; <https://wisqars.cdc.gov:8443/nvdrs/nvdrsDisplay.jsp>

2. Bureau of Labor Statistics (bls.gov). [Data file];2016. Available from <http://data.bls.gov/timeseries/LNU04000000?years_option=all_years&periods_option=specific_periods&periods=Annual+Data>; http://data.bls.gov/timeseries/LNS11300000

3. Centers for Disease Control and Prevention (CDC). WISQARS Web-based Injury Query and Reporting System, [Data file]; 2016. Available from http://www.cdc.gov/injury/wisqars/; <http://webappa.cdc.gov/sasweb/ncipc/mortrate10_us.html>

4. Eliason S. Murder-suicide: a review of the recent literature. J Am Acad

Psychiatry Law. 2009; 37: 371-376.

5. Marzuk, P., Tardiff, K. and Hirsch, C. "The epidemiology of murder-suicide." JAMA*.*1992; 267: 3179–3183.

6. Cohen, D., Llorente, M. and Eisdorfer, C. "Homicide-suicide in older persons." Am. J. Psychiatry. 1998; 155: 390-396.

7. Hannah, S.G., Turf, E.E. and Fierro, M.F. "Murder-suicide in central Virginia: a descriptive epidemiologic study and empiric validation of the Hanzlick-Koponen typology." Am. J. Forensic Med. Pathol. 1998;19: 275–283.

8. Hanzlick, R. and Koponen, M. "Murder-suicide in Fulton County Georgia 1988–1991: comparison with a recent report and proposed typology." Am. J. Forensic Med. Pathol. 1994;15: 168–173.

9. Campanelli, C. and Gilson, T. "Murder-suicide in New Hampshire, 1995–2000." Am.J. Forensic Med. Pathol. 2002; 23: 248–251.

10. Comstock, R.D., Mallonee, S., Kruger, E., Rayno, K., Vance, A. and Jordan, F. "Epidemiology of homicide-suicide events: Oklahoma, 1994–2001." Am. J. Forensic Med. Pathol. 2005; 26: 229–235.

11. Bossarte, R.M., Simon, T.R. and Barker, L. "Characteristics of homicide followed by suicide incidents in multiple states, 2003–2004." Inj. Prev. 2006; 12(2): ii33–ii38.
