# Supplementary material for: Suicides as a response to adverse market sentiment (1980-2016)
Source: PLoS One. 2017 Nov 2;12(11):e0186913. doi: 10.1371/journal.pone.0186913 (PMC5667934; doi:10.1371/journal.pone.0186913)

**S1 Figure**. Unemployment Rate versus Suicide Rate (1980-2016*). The correlation of these series is 0.62 (p-value <0.001). The suicide rate climbed in the 2005-2014 period to about 12.25 compared to 10.75 in the prior five years. During 2007-2010 the unemployment rate went up from about 5.1% to 9.6%. Since 2007, the Labor force participation rates have been dropping steadily, a relatively new phenomenon, from about 66% to 63% in 2016, resulting in a drop in the unemployment rate. The hardship for those who have given up on job searches possibly manifests in the steadily climbing suicide rate, post 2010. Data from CDC [1], BLS time series [2]: LNS11300000. **data available is only up to 12/2014, but as of 10/2016*.

**Unemployment Rate versus Suicide Rate (1980-2016*)**
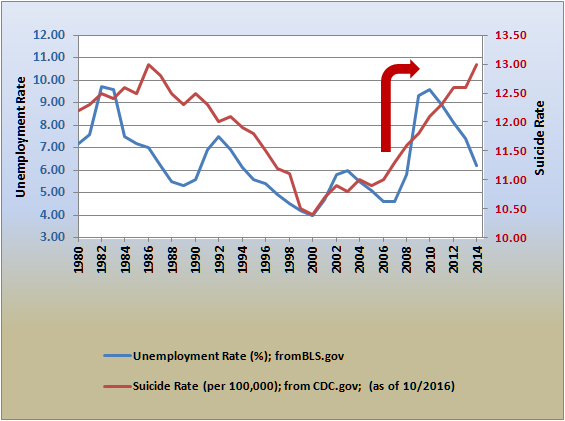

Supplement: S1 Fig — The correlation of these series is 0.62 (p-value <0.001). The suicide rate climbed in the 2005–2014 period to about 12.25 compared to 10.75 in the prior five years. During 2007–2010 the unemployment rate went up from about 5.1% to 9.6%. Since 2007, the Labor force participation rates have been dropping steadily, a relatively new phenomenon, from about 66% to 63% in 2016, resulting in a drop in the unemployment rate. The hardship for those who have given up on job searches possibly manifests in the steadily climbing suicide rate, post 2010. Data from [10], [9]: timeseries: LNS11300000, (*data available is only up to 12/2014, but as of 10/2016). (DOCX) [file pone.0186913.s002.docx]
