# Supplementary material for: Suicides as a response to adverse market sentiment (1980-2016)
Source: PLoS One. 2017 Nov 2;12(11):e0186913. doi: 10.1371/journal.pone.0186913 (PMC5667934; doi:10.1371/journal.pone.0186913)
Supplement: S2 Fig — Since 2007, the labor force participation rates have been dropping steadily, a relatively new phenomenon, from about 66% to 63% in 2016, resulting in a drop in the unemployment rate. The hardship for those who have given up on job searches possibly manifests in the steadily climbing suicide rate, post 2010. Data from [9]: timeseries:LNS11300000. (DOCX) [file pone.0186913.s003.docx]

**S2 Figure**. Declining Labor Force Participation. Since 2007, the labor force participation rates have been dropping steadily, a relatively new phenomenon, from about 66% to 63% in 2016, resulting in a drop in the unemployment rate. The hardship for those who have given up on job searches possibly manifests in the steadily climbing suicide rate, post 2010. Data from BLS *timeseries* [2]*:LNS11300000*.
