# Supplementary material for: Suicides as a response to adverse market sentiment (1980-2016)
Source: PLoS One. 2017 Nov 2;12(11):e0186913. doi: 10.1371/journal.pone.0186913 (PMC5667934; doi:10.1371/journal.pone.0186913)

**S3 Figure.**

OLS Regression Model: Suicide Rate. **SUIC_t_ = *φ*(UNEMP_t-2_, INFL_t-1_, GDP_t-1_, e_t_ )** This figure shows two aspects of the OLS regression with the CDC provided annual suicide rates as the dependent variable (all p-values are significant at the 0.015 level, Table1). The first exhibit indicates a positive relationship between the lagged national unemployment rate and the incidence of suicides. A 95% ellipse is also shown around the x-y points. The second exhibit shows the estimated versus the actual suicide rates. The observed adjusted R^2^ of this regression is 69.93%. Annual rates are since 1980. From [1], [2], [3].


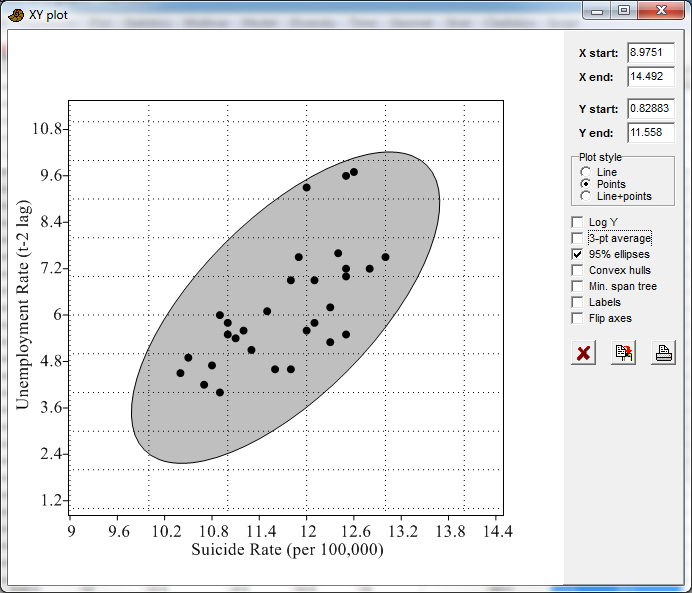

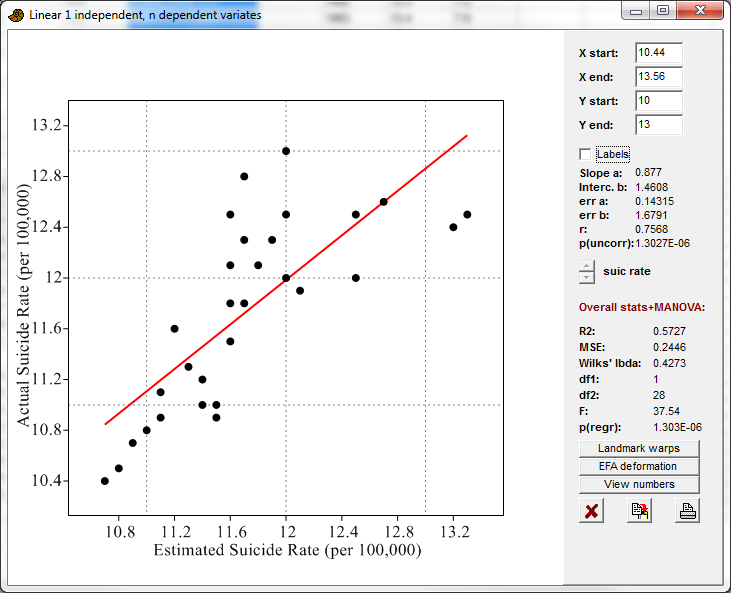

Supplement: S3 Fig — SUICt = φ(UNEMPt-2, INFLt-1, GDPt-1, et) This figure shows two aspects of the OLS regression with the CDC provided annual suicide rates as the dependent variable (all p-values are significant at the 0.015 level, Table 1). The first exhibit indicates a positive relationship between the lagged national unemployment rate and the incidence of suicides. A 95% ellipse is also shown around the x-y points. The second exhibit shows the estimated versus the actual suicide rates. The observed adjusted R2 of this regression is 69.93%. Annual rates are since 1980. From [9], [10], [25]. (DOCX) [file pone.0186913.s004.docx]
