# Supplementary material for: Suicides as a response to adverse market sentiment (1980-2016)
Source: PLoS One. 2017 Nov 2;12(11):e0186913. doi: 10.1371/journal.pone.0186913 (PMC5667934; doi:10.1371/journal.pone.0186913)
Supplement: S1 Table — Compiled and processed from [21] (*data available is only up to 12/2014, but as of 10/2016). (DOCX) [file pone.0186913.s005.docx]

**S1 Table**. Distribution of 2.79 Million U.S. Injury-Related Deaths (1999-2016*). Compiled and processed from the CDC/WISQARS database from the National Center for Injury Prevention and Control [3]. **data available is only up to 12/2014, but as of 10/2016*.

|  | **Injury Deaths**  **(1999-2014)** | **Percentage of Total Injury Deaths** | **Firearm Use** |
| --- | --- | --- | --- |
| **Unintentional Transportation** related deaths (motor vehicle related = 94%, excludes air, water, space, rail, bikes etc.) | 690,228 | 25% | - |
| **Suicides** | 560,632 (*509/day*) | **20%** | 52% |
| **Homicides** | 284,340 (*102/day*) | **10%** | 67% |
| **Unintentional non-Transportation** deaths (in declining order: Poisoning/Drugs, Falling, Fire, Suffocation, Drowning, Environmental, Firearms, Machinery, Cutting etc.) | 1,119,818 | 40% |  |
| **Undetermined**/Unclassified | 136,048 | 5% |  |
| **Total injury related deaths (**cumulative 1999-2014) | 2,791,066 | 100% |  |
