# Supplementary material for: Suicides as a response to adverse market sentiment (1980-2016)
Source: PLoS One. 2017 Nov 2;12(11):e0186913. doi: 10.1371/journal.pone.0186913 (PMC5667934; doi:10.1371/journal.pone.0186913)
Supplement: S2 Table — The sixteen states participating in the NVDRS (of CDC) are Alaska, Colorado, Georgia, Kentucky, Maryland, Massachusetts, New Jersey, New Mexico, North Carolina, Oklahoma, Oregon, Rhode Island, South Carolina, Utah, Virginia, and Wisconsin. Note the higher incidence rates for joint murder-suicides in 2009. Murder-Suicide is a separate crime class by itself and is not the sum of murders and suicides. Note the higher than average incidence rates for the years 2009 and 2010 [21] (*data available is only up to 12/2013, but as of 10/2016). (DOCX) [file pone.0186913.s006.docx]

**S2 Table**. Murder-Suicide (a joint event) data from National Violent Death Reporting System (2005-2016*). The sixteen states participating in the NVDRS (of CDC) are Alaska, Colorado, Georgia, Kentucky, Maryland, Massachusetts, New Jersey, New Mexico, North Carolina, Oklahoma, Oregon, Rhode Island, South Carolina, Utah, Virginia, and Wisconsin. Note the higher incidence rates for joint murder-suicides in 2009. Murder-Suicide is a separate crime class by itself and is not the sum of murders and suicides. Note the higher than average incidence rates for the years 2009 and 2010 [3] **data available is only up to 12/2013, but as of 10/2016*.
