# Supplementary material for: Suicides as a response to adverse market sentiment (1980-2016)
Source: PLoS One. 2017 Nov 2;12(11):e0186913. doi: 10.1371/journal.pone.0186913 (PMC5667934; doi:10.1371/journal.pone.0186913)
Supplement: S3 Table — Eliason [2] presents information on studies that report the incidence of 640 joint murder-suicides over the period 1980–2004. This study includes an additional 1680 such deaths for the period 2005–2013 (** available as of 10/2016). (DOCX) [file pone.0186913.s007.docx]

**S3 Table**. Overview of Previous Studies on Murder-Suicide^*^. Eliason [4] presents information on studies that report the incidence of 640 joint murder-suicides over the period 1980-2004. This study includes an additional 1680 such deaths for the period 2005-2013. **data* *available as of 10/2016*.

| **Study** | **Reported Incidence per 100,000 people** | **Murder-Suicide Incidents, *n*** | **Date Range** | **Location** |
| --- | --- | --- | --- | --- |
| [5] | 0.2– 0.3* | 11 incidents; 22 deaths | 1 week in 1989 | United States |
| [6] | 0.3– 0.7 in < 55-year age group* | 89 incidents in < 55-year group | 1988 –1994 | Southeast and West Central |
| [7] | 0.34 and 0.38* | 53 incidents; 116 deaths | 1980 –1984; 1990 –1994 | N/A |
| [8] | 0.46* | 12 incidents; 26 deaths | 1988 –1991 | Fulton County, Georgia |
| [9] | 0.26* | 16 incidents | 1995–2000 | New Hampshire |
| [10] | 0.3* | 73 incidents; 162 deaths | 1994 –2001 | Oklahoma |
| [11] | 0.23* | 65 deaths | 2003 | NVDRS (multiple states) |
| [11] | 0.238* | 144 deaths | 2004 | NVDRS (multiple states) |
| *This study* | *0.23** | *1,680 deaths *** | *2005* – *2013* | *NVDRS (all 16 reporting states)* |
|  |  |  |  |  |
| **1980-2013** | **Range 0.20 to 0.70** | **Total**  **murder-suicides 2,320** | **1980 -2013** | **data available as of 10/2016* |
| * per 100,000 of population | ** event where murder is followed by suicide, S2 Table |  |  |  |
